# FACTORS INFLUENCING EPISODIC MEMORY IN SUBJECTIVE COGNITIVE DECLINE: AN IMPLICATION FOR DEMENTIA PREVENTION

**DOI:** 10.1093/geroni/igz038.3255

**Published:** 2019-11-08

**Authors:** Sangwoo Ahn

**Affiliations:** University of Tennessee College of Nursing, Knoxville, Tennessee, United States

## Abstract

Episodic memory is typically affected early in the process of Alzheimer’s disease. Little is known about factors affecting episodic memory in subjective cognitive decline (SCD). The purpose of this study was to identify vascular and neuropsychiatric risk factors associated with episodic memory changes in older adults with SCD. Using the National Alzheimer’s Coordinating Center-Uniform Data Set, the relationship between baseline modifiable risk factors and episodic memory changes was analyzed using linear mixed-effects regression models. The study included a total of 1,401 subjects with SCD (mean ages: 74.0±8.2 years, 67.5% females, 84.2% White, mean follow-up period: 4.1±2.4 years). In univariate adjusted model, statistically significant coefficients on main effect or interaction with time were selected and entered into multivariate model, which was adjusted mutually for chosen independent variables and for all covariates. Reference in the final model was subjects without 1) hypercholesterolemia, 2) cigarette smoking history, and 3) depression. Those with hypercholesterolemia and former smokers had 0.024 and 0.035 points higher episodic memory scores than reference at baseline with similar rate of score changes between each group and reference over time, respectively. Current smokers scored 0.081 points lower than reference at baseline with similar rate of change over time between groups. Despite no difference at baseline, the score of depressed subjects decreased by 0.014 points a year compared to reference. It is important to manage current smoking and depression for older adults with SCD. Further research needs to identify which levels of cholesterol and smoking have a protective effect on episodic memory.

